# Updated Methods for Seed Shape Analysis

**DOI:** 10.1155/2016/5691825

**Published:** 2016-04-13

**Authors:** Emilio Cervantes, José Javier Martín, Ezzeddine Saadaoui

**Affiliations:** ^1^IRNASA-CSIC, Apartado 40, 37008 Salamanca, Spain; ^2^Regional Station of Gabes, Laboratory GVRF, INRGREF, University of Carthage, BP 67, Mnara, 6011 Gabès, Tunisia

## Abstract

Morphological variation in seed characters includes differences in seed size and shape. Seed shape is an important trait in plant identification and classification. In addition it has agronomic importance because it reflects genetic, physiological, and ecological components and affects yield, quality, and market price. The use of digital technologies, together with development of quantification and modeling methods, allows a better description of seed shape. Image processing systems are used in the automatic determination of seed size and shape, becoming a basic tool in the study of diversity. Seed shape is determined by a variety of indexes (circularity, roundness, and *J* index). The comparison of the seed images to a geometrical figure (circle, cardioid, ellipse, ellipsoid, etc.) provides a precise quantification of shape. The methods of shape quantification based on these models are useful for an accurate description allowing to compare between genotypes or along developmental phases as well as to establish the level of variation in different sets of seeds.

## 1. Introduction

There is a startling diversity of seed size and shape among the plant species all over the world. Seed size ranges from the dust seeds of the Orchidaceae and some saprophytic and parasitic species of about half to one millimeter in length to the massive sizes of coconuts in the Arecaceae family, for example,* Lodoicea maldivica* (J. F. Gmel.), reported to be the largest seeds in the world [1]. Quantitative evaluation of the shapes of biological organs is often required in various research fields, such as agronomy, genetics, ecology, and taxonomy [2]. Seed morphology has been useful for the analysis of taxonomic relationships in a wide variety of plant families and genus. Therefore both seed shape and size are useful parameters to analyze biodiversity in plants.

Seed morphology is useful in genotype discrimination [3] and the results are of significance in systematics. Measures of size and shape in seeds, their correlation, and relationship are important in breeding for seed yield [4]. Knowledge of the relation between seed shape and agronomic characteristics may be useful to improve yield or quality [5]. Biomorphological seed features may be analyzed by computer-aided image analysis systems and data quickly processed and stored in the hard disk, plotted or statistically elaborated [6]. Digital imaging can be a fast and reliable method for variety discrimination [3].

In this review, we focus on the parameters used to describe seed shape. The use of computer programs applied to digital images allows to obtain several indices useful to describe in detail the shape of the seed as well as to ascertain the level of variability. In addition, we discuss the use of these tools to taxonomical and genetic studies in diverse plant families.

## 2. Methods for Shape Analysis

Morphometry (from Greek “morphé,” meaning “shape” or “form,” and “metría,” meaning “measurement”) is the quantitative measurement of shape. The shape of the seed is interpreted by different methods involving several traits and diverse indices.

**Figure 1:**
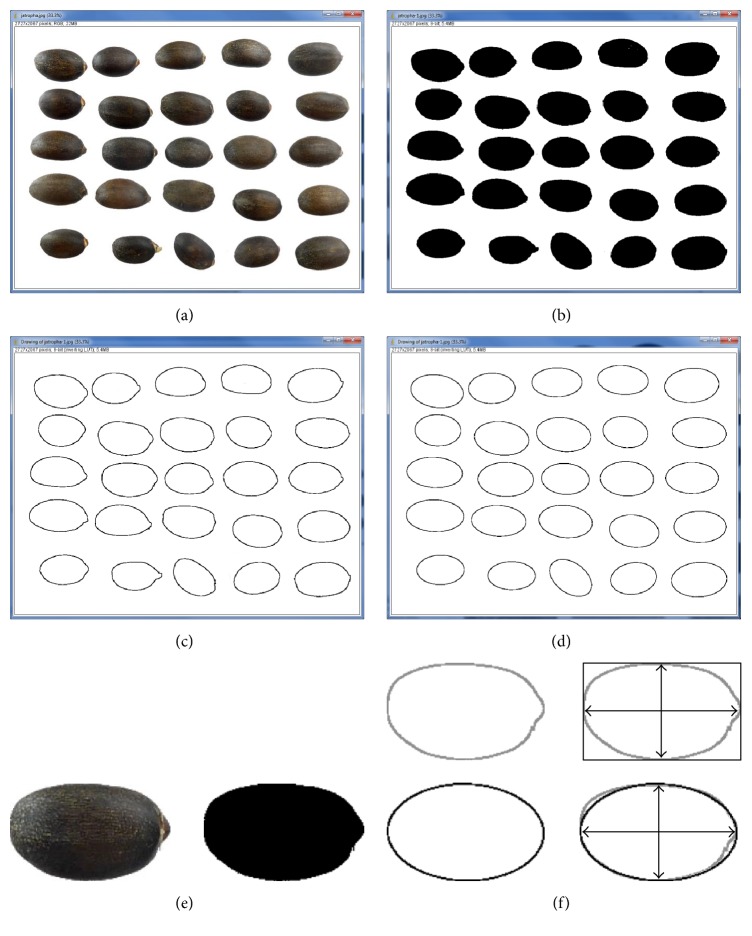
Digital processing of* Jatropha curcas* L. seed images. (a) Images corresponding to 25 seeds. (b) Binary images (black and white) of the seeds obtained by segmentation of the figure. (c) Silhouettes of seed images. (d) Ellipses adjusted to each seed image (fitted ellipses are given by the program Image J). (e) A seed with its image after segmentation and the silhouettes of the image (top) and the adjusted ellipse. (f) An example of the seed with its bounding rectangle (top) and the seed with the fitted ellipse, showing in both cases the major and minor axes. The values of area and perimeter, length, and width are obtained directly with Image J. The values are used to obtain the shape descriptors. The comparison of the area of the seed with the area of a model ellipse is used in the calculation of* J* index.* J* index is the ratio of shared (area common between seed and ellipse)/unshared area (see the text and Figure 2).

Technically, the data for shape analysis may be obtained in two ways: manual and computational. The simplest way is to measure seed length and width with calipers. However, manual methods have limits to the number of data, the quality of measurements, and the variety of shape data that can be generated and processed. By contrast, computational methods using digital imaging technology enable to measure automatically a variety of shape parameters at very small sizes in high-resolution images of large populations (Figure 1) [11].

In general, seed shape can be scored as a combination of magnitudes, or by a single magnitude that indicates the percentage of similarity to a given geometric object. We will describe the operations used in examples involving both cases.

Seed shape can be determined by the length/width ratio. Though not giving an accurate description of the seed shape, it is the simplest index to estimate and frequently used by many authors [12]. Balkaya and Odabas [13] refer to this magnitude as the Eccentricity Index (EI):

(1) $$\begin{array}{c} EI=\frac{L}{W}. \end{array}$$

Eccentricity Index is related with the aspect ratio (Image J), [14]. The aspect ratio of the particle's fitted ellipse is given by

(2) $$\begin{array}{c} AR=\frac{Major\;\;Axis}{Minor\;\;Axis}. \end{array}$$

Flatness Index (FI) is based upon the relationship between the particle dimensions along the three principal axes. It was developed by Cailleux [15] and it is used by Cerdà and García-Fayos [16] to characterize seed shape. The index is given by

(3) $$\begin{array}{c} FI=\frac{\left(L+W\right)}{2H}, \end{array}$$

where *L*, *W*, and *H* are the length, width, and height of the seeds, respectively. It ranged from a value of 1 for spheres to values greater than 2 for spindly seeds. For Thompson et al. [17] shape is related to seed length, width, and height, but this is still incomplete and other shape descriptors may be more precise.

The following shape descriptors are useful.

(1) Circularity index [18–20] or form factor is as follows [21]:
  (4) $$\begin{array}{c} I=\frac{4\pi \times area}{{perimeter}^{2}}. \end{array}$$

This index (*I*) is a measure of the similarity of a plane figure to a circle. It ranges from 0 to 1 giving the value of 1 for circles and it is a useful magnitude as a first approximation to seed shape. In figures having many small protuberances through the surface, the perimeter increases and circularity index has lower values. In these instances it is advisable to use roundness, because this magnitude is independent of such perimeter irregularities.

(2) Roundness [14] is
  (5) $$\begin{array}{c} R=\frac{4\times area}{\pi \;\;{\left[Major\;\;axis\right]}^{2}}. \end{array}$$
(3) Rugosity or roughness is defined as the ratio of the perimeter to the convex perimeter [22]:
  (6) $$\begin{array}{c} I=\frac{P_{s}}{P_{c}}, \end{array}$$

where *P*_*s*_ is the perimeter of the seed and *P*_*c*_ is the convex perimeter of the seed, also known as convex hull, that is, the smallest convex figure that contains all the points of an image.

## 3. Seed Shape Analysis Based on Diverse Indexes

The work of Vijaya Geetha et al. [23] with mustard genotypes uses the shape factor as a descriptor. This allows the comparison between genotypes and the grouping by similarity in clusters.

Kara et al. [24] used image analysis system for the description and classification according to seed size and shape of twelve different common bean (*Phaseolus vulgaris* L.; Fabaceae) cultivars. Their work includes diverse magnitudes such as area, sphericity, and shape factor allowing to determine the relationships among the bean cultivars.

## 4. Shape Analysis by Comparison with Geometric Figures:* J* Index

**Figure 2:**
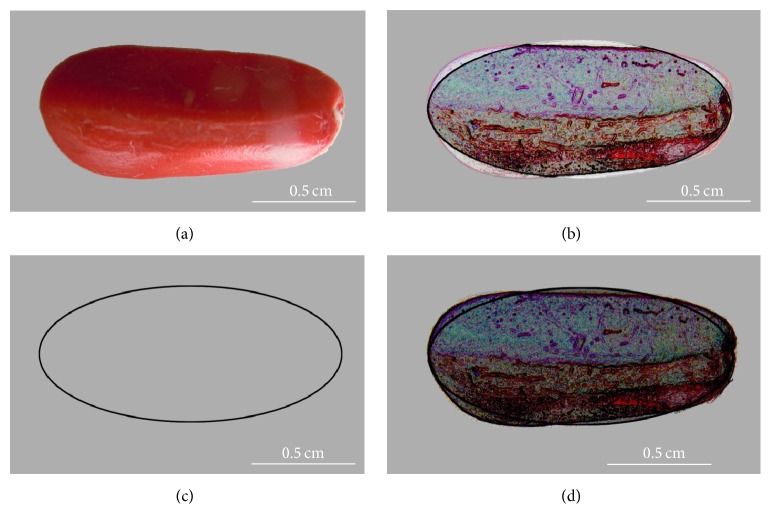
The image represents a seed of* Magnolia* sp. (a) and the ellipse (c). Shared regions are represented in (b) and the total (shared plus nonshared) in (d). *J* index is the ratio between shared regions and the total: *J* = (area  (C)/(area  (C) + area  (D))) × 100, where C represents the common region and D the regions not shared. Scale bar represents 0.5 cm.

**Figure 3:**
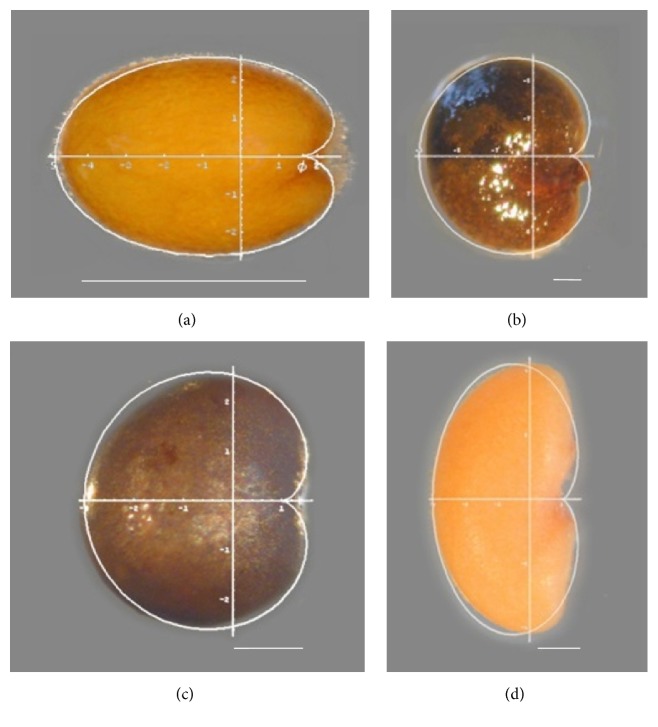
Cardioid or cardioid derived models applied in (a)* Arabidopsis thaliana* seeds, a cardioid elongated in the *x*-axis for a factor of Phi (1,618) [7]; (b)* Capparis spinosa*, a cardioid [8]; (c)* Lotus japonicas*, a cardioid [9]; and (d)* Medicago truncatula*, a cardioid curve elongated in the *y*-axis for factor of Phi [9]. Scale bar represents 0.5 mm.

**Figure 4:**
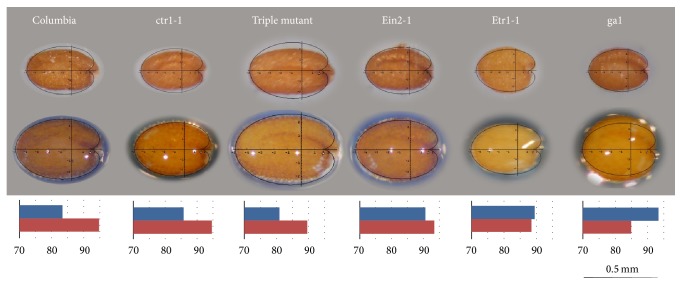
Dry seeds (top) and seeds imbibed during 1 h (middle) of Columbia,* ctr1-1*,* etr1-1*, and* ga1-1* mutants. Graphics show the values of *J* index in dry seeds (above) and imbibed seeds (below). Triple mutant is (*ein2-1*,* etr1-7*, and* ers1-2*) [10]. Scale bar represents 0.5 mm.

**Figure 5:**
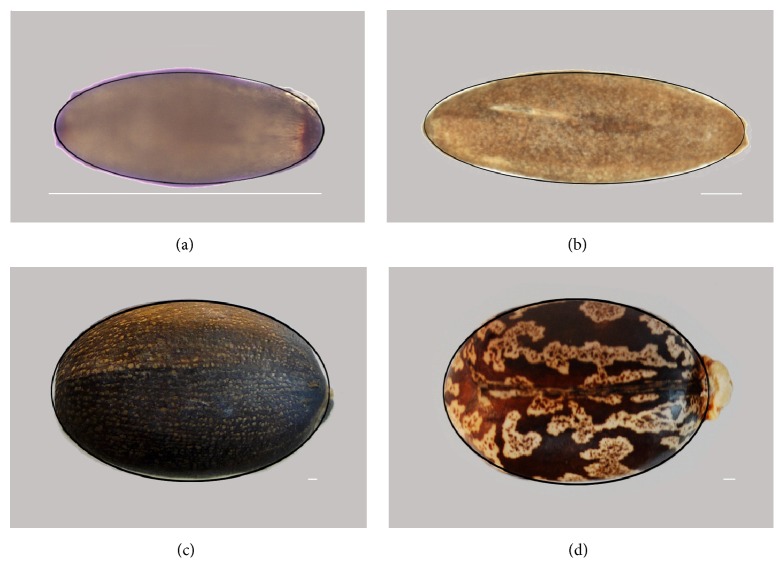
Seed shape models based on ellipses may be used for Campanulaceae and Apocynaceae ((a)* Campanula dichotoma* L.; (b)* Nerium oleander* L.) and have been applied in the description of Euphorbiaceae seeds ((c) and (d) correspond to seeds of* Jatropha curcas* L. and* Ricinus communis* L.). Scale bar represents 0.5 mm.

**Figure 6:**
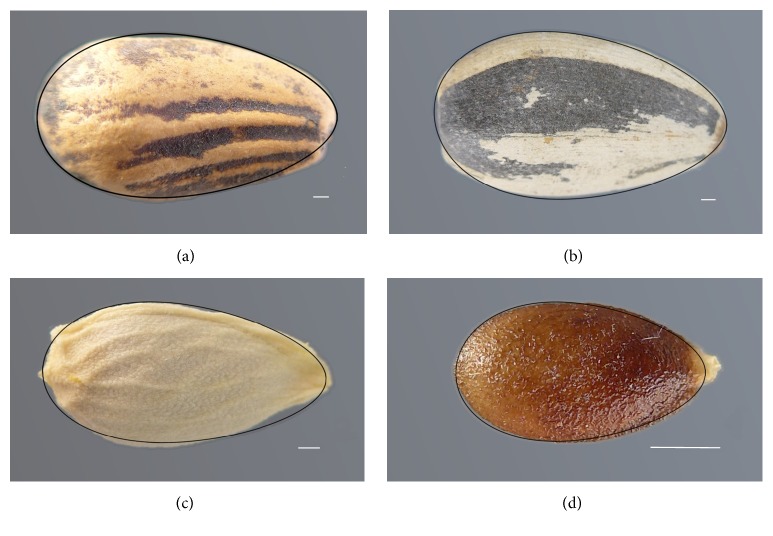
Seed shape models based on the ovoid may be used for the Pinaceae ((a)* Pinus pinea* L.), Asteraceae ((b)* Helianthus annuus* L.), Rutaceae ((c)* Citrus reticulata* Blanco), and Cucurbitaceae ((d)* Ecballium elaterium* L.). Scale bar represents 1 mm.

**Figure 7:**
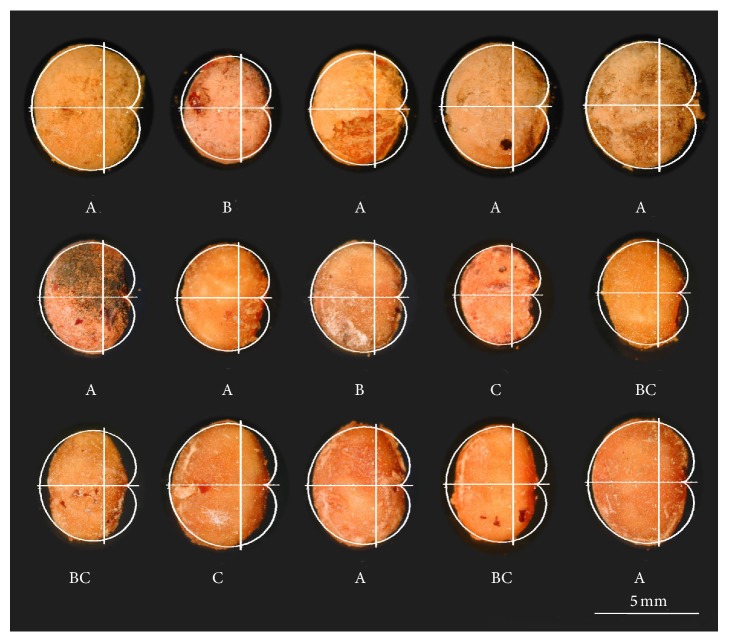
A model based on the cardioid was applied to seeds of* Rhus tripartita* (Ucria) Grande (Anacardiaceae; Saadaoui et al., submitted). Four morphological types were described in this work. Type A: seeds in which similarity with the cardioid is above 92 in the left region and above 80 in the right. Type B: seeds whose values of similarity with the cardioid curve are below 92 in the left region and above 80 in the right of the seed. Type C: seeds whose values of similarity with the cardioid curve are below 80 in the right part of the seed and above 92 in the left. Type BC: seeds in which similarity with the cardioid curve is below 92 per cent in the right and below 80 per cent in the right part of the seed. Plants grown in different climates had distinct proportions of seed types.

Description of seed shape using a single nondimensional magnitude is based on the percentage of similarity to a given geometric object. Seed images are compared with geometric figures taken as models (Figures 2–7). Modeling based on geometric figures contributes to increased precision in the quantification of seed shape allowing to determine morphological variation, including changes in the course of imbibition, alterations in mutants, differences between related genotypes, or changes in shape in response to environmental factors.

*Arabidopsis thaliana* (L.) Heynh. (Cruciferae) is a useful plant model for studying seed development due to its ease of cultivation and extensive genetic and community resources available. Similar to size analysis [25], shape analysis in the model plant* A. thaliana* may be a basic tool to investigate the coordinate metabolic pathways that regulate seed development.

Cardioid-based figures were found accurate in the shape modeling of* Arabidopsis thaliana* seeds. Cervantes et al. [7] used a cardioid elongated in the *x*-axis for a factor of Phi as a model to obtain a magnitude representing the shape of seeds in* Arabidopsis thaliana*: the* J* index. Phi is the Golden Ratio and its value is approximately 1,618. To obtain the* J* index (Figure 2), the areas in two regions were compared: the regions shared by the cardioid and the seed image (common region, C) and the regions not shared between both areas (D). The index of adjustment (*J*) is defined by

(7) $$\begin{array}{c} J=\frac{area\;\;\left(C\right)}{area\;\;\left(C\right)+area\;\;\left(D\right)}\times 100, \end{array}$$

where C represents the common region and D the regions not shared. Note that* J* is a measure of seed shape, not of its area. It ranges between 0 and 100 decreasing when the size of the nonshared region grows and equals 100 when cardioid and seed image areas coincide; that is, when area (D) is zero.

In* Arabidopsis thaliana*, Martín et al. [26] compared seed shape during the sustained period of seed imbibition in wild-type and mutant seeds and observed differences during imbibition between wild-type and seeds mutant in cellulose biosynthesis and ethylene perception and response. Seed shape was compared essentially by* J* index (Figure 4). A maximum value of* J* index is observed in the first minutes after water contact within the seed. In the course of imbibition the seeds tend to adopt the shape of the geometric model and* J* index reaches values over 95.

The cardioid figure was applied also in the model legumes* Lotus japonicus* (Regel) K. Larsen and* Medicago truncatula* Gaertn., whose seeds look like a cardioid curve (*Lotus japonicus* (Regel) K. Larsen; Figure 3(c)), or a cardioid curve elongated in the *y*-axis for factor of Phi (*Medicago truncatula* Gaertn.) [9], as well as to analyze differences between two subspecies of* Capparis spinosa* L. (Capparaceae; Figure 3(b)) [8]. The models proposed allow the comparison between genotypes (species, varieties, or mutants), or treatments, as well as diverse phases of growth [26, 27]. A model based on the cardioid was also applied to seeds of* Rhus tripartita* (Ucria) Grande (Anacardiaceae; Saadaoui et al. submitted; Figure 7).

Ellipses have been applied as models in the description of seed shape in the Euphorbiaceae (Figure 5) [28] and may be also applied to the Poaceae. Ovoids or modified ovoids can be good models for the Asteraceae and the Cucurbitaceae (Figure 6).

Other methods may be applied for taxa in which seed shape does not adjust well to a geometric figure. Elliptic Fourier Descriptors (EFDs) can delineate any type of shape with a closed two-dimensional contour and have been effectively applied to the evaluation of various biological shapes in animals and plants. Quantization of shapes is a prerequisite for evaluating the inheritance of morphological traits in quantitative genetics. There are many reports showing that measurements based on EFDs are helpful for such quantization of the shapes of plant and animal organs [2].

## 5. Studies of Shape Based on Cardioid Models in Diverse Plant Families

Seed image analysis based on geometric models may contribute to the botanical description of species, genus, or families and the identification and discrimination of genotypes, varieties, and species and the determination of diversity at inter- and intraspecific levels.

### 5.1. Brassicaceae

The comparison of* Arabidopsis thaliana* seed images with the cardioid gave values of* J* index close to 90 and over 95 in the course of imbibition (Figure 3(a)). Mutants in the ethylene response pathway* etr1-1* had reduced values of* J* index (Figure 4) [7, 26], and similar results were observed in cellulose biosynthesis mutants [26, 27].* J* index provides thus a tool for the rapid phenotyping of seeds. It may be interesting to evaluate* J* index in other species of* Arabidopsis*, as well as in massive screens of mutants or genetic variations to identify the nucleotide sequences and functions related with seed shape.

### 5.2. Fabaceae

Gandhi et al. [29] used seed morphological and micromorphological features to study 17 legume species belonging to three genera* Crotalaria*,* Alysicarpus,* and* Indigofera*, of Faboideae, Fabaceae. The study involves grouping of seeds in morphological types such as oblong, ovoid, ellipsoid, orbicular, and reniform (elongated cardioid, also sometimes called* kidney shaped*). Turki et al. [30] examined seed morphology of nineteen species of the genus* Trigonella* (Fabaceae) and found variation in the shape of species; four types of seed were recognized: elliptic, rhomboid, ovoid, and rectangular. Description of shape requires an accurate quantification and these studies may benefit from the comparison with cardioid models.

Our work with the model legumes* Lotus japonicus* (Regel) K. Larsen and* Medicago truncatula* Gaertn. showed similarity between the seed images and the cardioid (*Lotus japonicus*; Figure 3(c)), or the seed images and a cardioid elongated in the *y*-axis for factor of Phi (*Medicago truncatula*; Figure 3(d)) [9]. In* Lotus japonicus* values of* J* index were superior to 90 in dry seeds for all genotypes considered and in the imbibed seeds ethylene insensitive mutants had reduced values of* J* index in relation to wild-type seeds. In* Medicago truncatula* values of* J* index in dry seeds were of 87.1 and 86.8 for wild-type (dry and imbibed seeds) and 86.0 and 86.4 for the sickle mutant (*etr1-1*). Thus,* J* index was lower in the sickle mutant (*etr1-1*).

### 5.3. Capparaceae

The Capparaceae family, in the order Brassicales, is related to the Brassicaceae.

*Capparis spinosa* seeds adjust well to a cardioid (Figure 3(b)). Saadaoui et al. [8] analyzed seed shape in two subspecies (*Capparis spinosa* subp.* spinosa* and* Capparis spinosa* subp.* rupestris*) and observed a relation between shape variation and subspecies: shape is more variable in* Capparis spinosa* subp.* rupestris*, but* Q*1 values expressing similarity to the cardioid in the first quadrant of the seed were reduced in* Capparis spinosa* subp.* spinosa*. These results support the hypothesis that the former is a primitive, nonspecialized subspecies with characteristics of an “r” type strategy. Fici [31] suggested that* Capparis spinosa* subp.* rupestris* represents a primitive type closer to the tropical stock of the group, whereas* Capparis spinosa* subp.* spinosa* is a derived form of this. In support of this idea,* Capparis spinosa* subp.* rupestris* has several characteristics of a plant with an “r” type strategy [32]: small seeds, simple structure (trailing, thornless), larger number of stamens, and self-reproduction. In contrast,* Capparis spinosa* subp.* spinosa* may have diverged from the “r” strategy towards more specialized adaptations: larger seeds, more complex structure (erect and thorny), reduced number of stamens, and cross-reproduction [33] as well as seeds with particular morphology (less varied and reduced values of* J* index in the first quadrant) [8].

### 5.4. Anacardiaceae

The Anacardiaceae is a complex family including trees and shrubs of diverse ecological significance and geographical distribution. The seeds of* Rhus tripartita* (Ucria) Grande are similar to the cardioid (Figure 7). Analysis of* J* index in nine natural populations of* Rhus tripartita* grown in Tunisia reveals values comprised between 76.2 and 95.3. Differences between populations were found both in size as well as in shape (circularity index,* J* index total, and partials). Morphological types were characteristic for some of the populations. Differences in shape are independent of size for this species (Saadaoui et al., submitted).

## 6. Studies of Shape Based on Ellipse Models

### 6.1. Euphorbiaceae

Morphological aspects of seeds in the genus* Euphorbia* have been studied in some detail. These include surface characters such as cellular arrangement, cell shape, relief of outer cell walls, and epicuticular secretions [34]. Morphological types have been associated with sections of the genus; thus ellipsoidal type is associated with section* Helioscopia*, ovoid-quadrangular with* Myrsinaceae,* and pseudo-hexahedral with* Herpetorrhizae* [35].

Seed shape quantification in* Jatropha curcas* L. was based on the comparison with an ellipse. The study of eight genotypes from Africa and America planted in the same field reveals diversity in seed shape. The seeds of cultivars with lower seed yield had reduced values of* J* index [28].

Also, seed shape of* Ricinus communis* L. is quantified with an ellipse based model. Although this species belongs to the Euphorbiaceae family, as* Jatropha curcas*, the comparison between seeds obtained from plants grown in diverse locations in Tunisia showed lower diversity in seed shape than observed in* J. curcas* (Martin et al., International Journal of Agronomy in the press). In agreement with the results of Gegas et al. [36] reported for* Triticum* (Poaceae), seed shape in* Ricinus communis* was also found to be independent of size.

### 6.2. Pinaceae

An ellipse is used as a model for seed shape in coniferous trees in the Pinaceae (Scots pine, European black pine, Norway spruce, and Stone pine; Figure 6(a)) and Taxaceae (*Taxus baccata* L.), whereas a double right quadrangular pyramid has been applied for silver fir and Douglas-fir seeds in the Pinaceae [37].

## 7. Seed Shape Regulation

### 7.1. Seed Shape Regulation in Model Plants

Individual genes encode functions directly related with seed shape. This may be the case in hormone synthesis, metabolism, or signaling pathways, as well as genes encoding structural components. In* Arabidopsis thaliana* we have indicated the effect of ethylene perception and cellulose synthase mutants on seed shape. In addition,* etr1-1* mutants also affect seed shape in the model legumes* Lotus* and* Medicago*.

Genetic analysis of a seed shape mutant of* Arabidopsis thaliana* isolated from an ethyl methane sulfonate-treated population revealed that the heart-shaped phenotype was maternally inherited, showing that this is a testa mutant. This indicated the importance of the testa for the determination of the seed shape. This recessive aberrant testa shape (*ats*) gene was located at position 59.0 on chromosome 5 [38].

In* Arabidopsis thaliana*, brassinosteroid (BR) plays crucial roles in determining the size, mass, and shape of seeds; the seeds of the BR-deficient mutant de-etiolated2 (det2) are smaller and less elongated than those of wild-type plants due to a decreased seed cavity, reduced endosperm volume, and integument cell length [39].

### 7.2. Seed Shape Regulation and Adaptation

Seeds consist of an embryo plus endosperm, plus a protective seed-coat or testa. Many seeds have distinctive dispersal appendages in the seed, such as plumes and hairs [1]. Seed morphology often indicates the general means of dispersal and shape is adapted for dispersal. Although variations in seed shape are classically interpreted almost wholly as adaptations for dispersal, some features of shape may be thrust upon a seed by the conditions inside the ovary in which it develops [40]. Liu et al. [41] examined 70 species from the cold Gurbantunggut Desert in northwest China and identified five dispersal syndromes (anemochory, zoochory, autochory, barochory, and ombrohydrochory). Barochorous species were significantly smaller and rounder than the others but did not find a correlation between seed shape and germination percentage.

In other instances seed shape can act on germination physiology. In maize, shape has an effect on seed physiological quality: seed germination, seed emergence, and speed of germination [42]. Gardarin and Colbach [43] studied 33 species and reveled that proportions of nondormant seeds were higher for elongated than spherical seeds.

Other factors which determine the final shape of the seed are climatic, for example, wind, rainfall, or humidity, and intrinsic characteristics of the mother plant, for example, height, ballistic mechanisms, and of course diaspore morphology [44]. In* Kohlrauschia prolifera* (L.) Kunth (Caryophyllaceae), three taxa differ in seed shape, but some variation is related to environmental gradients [40]. Seed shape and size act in seed removal by the surface wash; seeds greater than 50 mg with spherical shapes were easily removed than flat shaped seeds [16]. Peco et al. [45] studied seed persistence in the soil for 58 abundant herbaceous species. Persistence is elevated in small seeds, but there is no relation between seed persistence and shape.

Donnelly et al. [46] studied seeds from two diploid subspecies of* Setaria viridis* (L.) P.Beauv. (Poaceae), consisting of one weedy subspecies and two races of the domesticated subspecies, and four other poliploid weedy species of* Setaria*. Three-dimensional models gave further evidence of differences in shape reflecting adaptation for environmental exploitation. The selective forces for weedy and domesticated traits have exceeded phylogenetic constraints, resulting in seed shape similarity due to ecological role rather than phylogenetic relatedness [46]. The transition between wild plant forms and domesticated species can be considered an evolutionary adaptation by plants in response to a human driven ecology; seeds tended to change shape and size under domestication [47].

## 8. Conclusion

Seed shape is one of the features discussed for seed description and the analysis of intra- and interspecific variability. The availability of software for digital image analysis helps with the development of several indices enabling the modeling of seed shape, according to virtual curves (cardioid, ellipse, circle, ovoid, etc.). This allows quantification of seed shape that can be used in comparative taxonomy, genetics, physiology, and biochemistry. Seed shape is influenced by genetic and environmental factors. It is related to the taxonomic status and may be, as well, related to the physiology of germination and yield of seed products (starch, fixed oils, protein, etc.).

The morphological description of plant structures is a requisite for understanding the relationships between structure and function in evolution and may contribute to defining developmental situations associated with genomic composition and activity. Changes in shape may be either the result of developmental programs in a “regular” environment or the response to changes (stress) in environmental conditions [48]. Modeling seed shape by geometric figures is an easy approximation that may help to understand and quantify morphological variation in seeds, changes in the course of imbibition, and alterations in mutants as well as differences between related genotypes. Analysis of seed shape has unexpected applications in botany and agrobiology.
